# Individual and Environmental Factors Influencing Adolescents’ Dietary Behavior in Low- and Middle-Income Settings

**DOI:** 10.1371/journal.pone.0157744

**Published:** 2016-07-22

**Authors:** Roosmarijn Verstraeten, Jef L. Leroy, Zuzanna Pieniak, Angélica Ochoa-Avilès, Michelle Holdsworth, Wim Verbeke, Lea Maes, Patrick Kolsteren

**Affiliations:** 1 Nutrition and Child Health Unit, Department of Public Health, Prince Leopold Institute of Tropical Medicine, Antwerp, Belgium; 2 Poverty, Health, and Nutrition Division, International Food Policy Research Institute (IFPRI), Washington, DC, United States of America; 3 Consumer and Sensory Research Institute Ltd (IBKiS), Warsaw, Poland; 4 Department of Agricultural Economics, Faculty of Bioscience Engineering, Ghent University, Ghent, Belgium; 5 Departamento de Biociencias, Universidad de Cuenca, Cuenca, Ecuador; 6 Public Health section, School of Health and Related Research (ScHARR), The University of Sheffield, Sheffield, United Kingdom; 7 Department of Public Health, Faculty of Medicine and Health Sciences, Ghent University, Ghent, Belgium; Leibniz Institute for Prevention Research and Epidemiology (BIPS), GERMANY

## Abstract

**Objective:**

Given the public health importance of improving dietary behavior in chronic disease prevention in low- and middle-income countries it is crucial to understand the factors influencing dietary behavior in these settings. This study tested the validity of a conceptual framework linking individual and environmental factors to dietary behavior among Ecuadorian adolescents aged 10–16 years.

**Methods:**

A cross-sectional survey was conducted in 784 school-going Ecuadorian adolescents in urban and rural Southern Ecuador. Participants provided data on socio-economic status, anthropometry, dietary behavior and its determining factors. The relationships between individual (perceived benefits and barriers, self-efficacy, habit strength, and a better understanding of healthy food) and environmental factors (physical environment: accessibility to healthy food; social environment: parental permissiveness and school support), and their association with key components of dietary behavior (fruit and vegetables, sugary drinks, breakfast, and unhealthy snack intake) were assessed using structural equation modeling.

**Results:**

The conceptual model performed well for each component of eating behavior, indicating acceptable goodness-of-fit for both the measurement and structural models. Models for vegetable intake and unhealthy snacking showed significant and direct effects of individual factors (perceived benefits). For breakfast and sugary drink consumption, there was a direct and positive association with socio-environmental factors (school support and parental permissiveness). Access to healthy food was associated indirectly with all eating behaviors (except for sugary drink intake) and this effect operated through socio-environmental (parental permissiveness and school support) and individual factors (perceived benefits).

**Conclusion:**

Our study demonstrated that key components of adolescents’ dietary behaviors are influenced by a complex interplay of individual and environmental factors. The findings indicate that the influence of these factors varied by type of dietary behavior.

## Introduction

Globally, 42 million children are overweight or obese—the consequence of a staggering 47.1 percent rise in prevalence between 1980 and 2013 [1]. A rise no longer exclusive to high-income countries as the prevalence of childhood and adolescent overweight and obesity has also reached alarmingly high levels in low- and middle-income countries (LMICs). In Latin-America 25 percent of children and adolescents are overweight or obese [2]. Nearly half of all overweight children under 5 years of age now live in Asia, and a further 25 percent are found in Africa [3, 4]. Poor dietary behavior is a key factor in the onset of obesity and an important contributor to the global disease burden [5]. Despite the accumulation of evidence illustrating unhealthy food practices among young people in LMICs [6–8], the determinants of their dietary behavior remains poorly understood.

Behavioral theories and conceptual frameworks have been recommended to identify and better understand influences on dietary behavior [9], but their utility for use in adolescents in LMICs is limited. Firstly, the majority of theories to date have been developed for American or European adults [10]; testing their validity for use in other cultures and local contexts, has rarely been undertaken [11, 12]. Furthermore, the age groups the models apply to have not been specified [13]. As such, they may neither be applicable nor transferable to young people living in LMICs. Secondly, much of what is known about the individual (e.g. self-efficacy and habit strength) and environmental (e.g. parental permissiveness and accessibility) factors influencing dietary behavior comes from qualitative studies [14, 15]. Few attempts have been made to use well-articulated, i.e. evidence- and theory-based, conceptual models to i) identify factors that adequately reflect the social and cultural reality of young people in LMICs [16–18] and ii) quantify the pathways and their strength by which individual and environmental factors interact and affect, both directly but also indirectly, dietary behaviors [19, 20].

A recent qualitative theory-based study we undertook in Ecuadorian adolescents showed that “healthy foods”, such as fruit and vegetables, were perceived as vital to healthful eating. This study resulted in a composite framework (evidence- and theory-based), in which eating behavior was conceptualized as the result of individual and environmental influences [21]. In the present study, we sought to further the evidence of this conceptual model by identifying and quantifying the relationships (direct and indirect) between factors and their influence on key components of dietary behavior. Our study focused on four components, fruit and vegetables, sugary drinks, breakfast, and unhealthy snack intake. They correspond with how adolescents viewed healthy eating [21] and reflect important problems with their current dietary behavior [7]. Furthermore, each of these components has been independently associated with a high risk of obesity and/or chronic diseases: high intakes of specific foods such as sugary drinks [22] and processed foods [23] has been associated with obesity and its related diseases and weight gain, respectively; erratic behaviours such as skipping breakfast has been shown to be associated with obesity [24]; and diets low in fruit and vegetables and whole grains, nuts and seeds, and seafood omega-3 fatty acids were shown to be associated with high risk of chronic diseases [5].

## Methods

### Design and study population

This study used data from a cross-sectional survey that was conducted in Ecuador from January 2008 to April 2009. Participants were 10–16 year old adolescents (*n* = 784) from an urban (Cuenca) and rural (Nabón) area in Ecuador. A different sampling frame was used for each area: all school-going children willing to participate were included in Nabón, while a two-stage cluster design was used (with schools as primary and classes as secondary sampling units) in Cuenca. Adolescents were excluded if they were pregnant, followed a special diet or suffered from a severe medical or physical disorder. A detailed description of the sample and study procedures is given elsewhere [25].

### Ethics, consent and permission

The study protocol was granted ethical approval from Ecuadorian (University Central in Quito; CBM/cobi-001) and Belgian (Ghent University Hospital; 2008/462—FWA00002482) Ethical Committees. Informed assent was obtained from all participants. Parents/guardians provided written informed consent.

### Measurements

Data were collected at school during class time by a research team extensively trained according to a predefined protocol and training manual.

#### Socio-demographic attributes

Data on age, gender (male/female), geographic location (urban/rural) and socio-economic status (SES) were collected. The latter was assessed using a method developed by the Integrated Social Indicator System for Ecuador [26], based on World Bank recommendations to develop household surveys in LMICs [27]. This method measures poverty using the “Unsatisfied Basic Needs” criteria and classifies a household as poor when it lacks access to one or more basic needs (such as education, health, nutrition, housing, urban services and employment opportunities). Using this method, participants were classified into two groups: “Poor” and “Better off”.

#### Anthropometric measurements

Anthropometric measurements were carried out in duplicate by two trained researchers. Adolescents wore light clothing but no shoes during the measurements. Height was measured to the nearest 0.1 cm with a portable stadiometer (model PORTROD, Health O Meter, USA) and body weight to the nearest 0.1 kg using a digital calibrated balance (model SECA 803, Seca GmbH & CO, Hamburg, Germany). Adolescents were then classified into age- and sex-specific Body Mass Index (BMI) categories (underweight, healthy weight, overweight and obese) according to the International Obesity Task Force criteria [28, 29].

#### Dietary behavior

Food intake was measured using two interview-administered 24h dietary recalls on a randomly selected weekday and weekend day. Local household measures (cups, bowls, etc.) were calibrated and used by the trained interviewers to quantify the amount of food consumed. A food composition database was compiled using databases from the US (USDA, 2012), Mexico (INNSZ, 1999), Central America (INCAP/OPS, 2012) and Peru (CENAN/INS, 2008). When detailed information on the ingredients and/or cooking methods of a recipe was unavailable, recipes were prepared in triplicate by local volunteers. The ingredients used, and their weights, were measured and averaged to obtain a final estimate for the recipe. For locally processed and pre-packed food items, food labels were used to obtain the food composition.

Data for the four components of dietary behavior were extracted from both 24h recalls. Fruit and vegetable intake were examined separately and combined. Sugary drinks included all soft drinks, fizzy drinks, energy drinks, and juices with added sugar. Breakfast was defined as a meal consumed between 5:00–7:00 am or 5:00–8:00 am for adolescents in schools with a morning or afternoon schedule, respectively. Unhealthy snacks were defined as foods rich in sodium, fat or sugar (e.g. sweets, salty snacks, and any other packaged food) eaten as a morning, afternoon or evening refreshment. Sugary drinks and fruit and/or vegetable intake were calculated as the total average daily intake (g/day) over both days to best represent habitual intake. Breakfast and intake from unhealthy snacks were expressed as a percentage of daily energy intake averaged over both days (E %/day).

#### Assessment of individual and environmental factors influencing dietary practices

**Fig 1:**
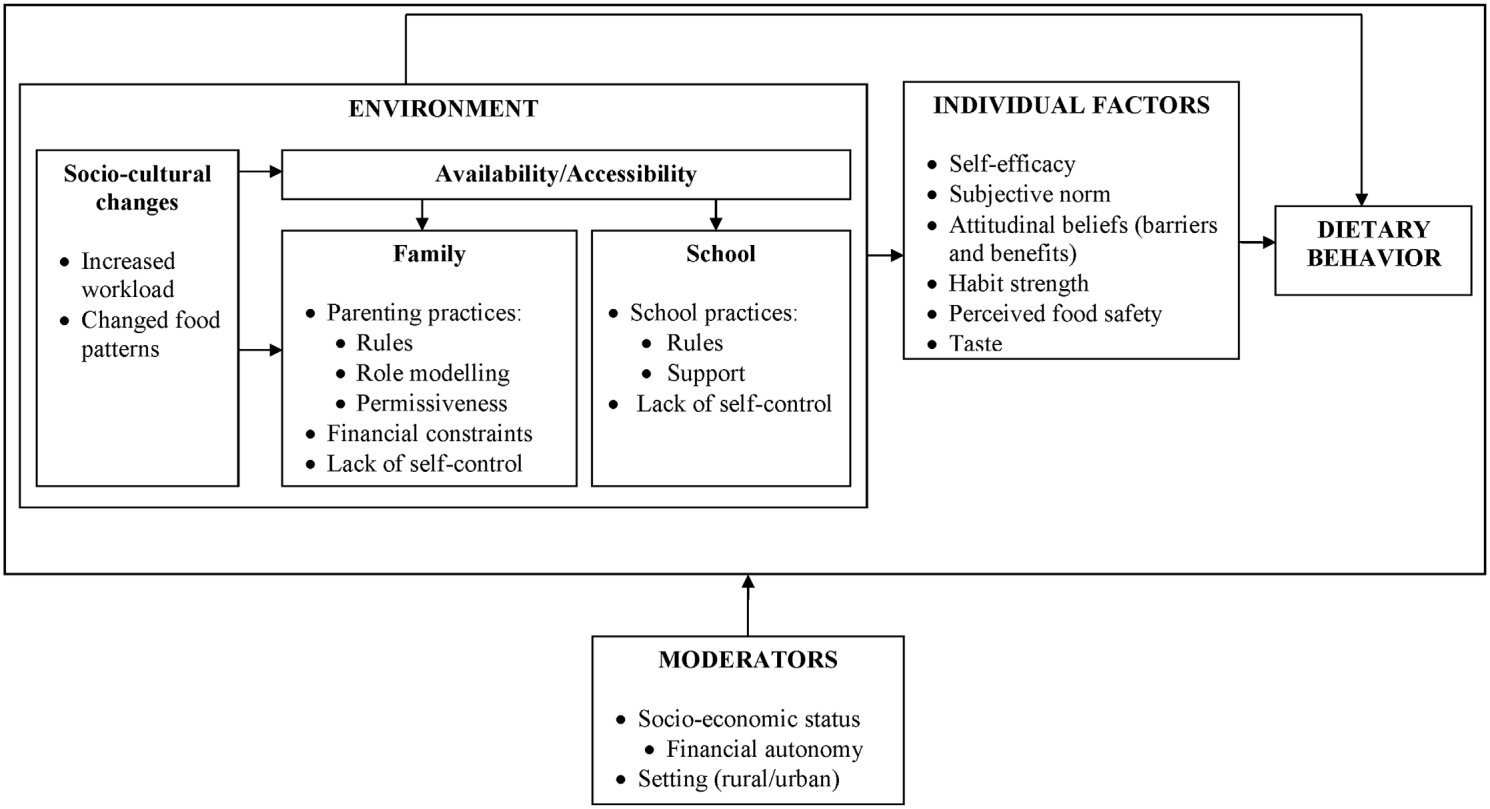
Conceptual framework for healthy dietary behavior in an Ecuadorian population.

The conceptual framework including key individual and environmental factors for dietary behavior is illustrated in Fig 1 [21]. A self-administered questionnaire was used to quantify each factor. As no culturally appropriate and validated psychometric scales to measure these factors existed, a questionnaire was developed using qualitative data from this population [21], relevant literature [30], and the expertise of the research team. The questionnaire was piloted for understanding and readability with a group of school-going adolescents (11–15 y old) not included in this study using cognitive interviewing (a qualitative process encompassing two main techniques: think aloud interviewing and verbal probing) [31]. As part of this pretesting, the questionnaire was administered twice with a four week interval. Both single and multiple items were used to measure factors (i.e. constructs) in the framework; socio-cultural changes and lack of self-control were not measured. Items in the questionnaire were measured using 5-point interval scales. Items were recoded into the same direction so that higher construct scores corresponded to the most favorable conditions for healthy dietary practices (e.g. a high score on perceived barriers indicates fewer barriers to eat healthily). Sum scores were calculated for each construct. The outcome variables were left unchanged.

### Statistical analysis

Anthropometric, socio-demographic and questionnaire data were entered in duplicate in Epidata (Version 3.14, Odense Denmark) by two researchers. Food intake was entered using an online software package designed for 24h dietary recalls (Lucille software 0.1, 2010, Ghent University; http://www.foodscience.ugent.be/nutriFOODchem/foodintake).

Data on food intake, anthropometry, socio-demographics, questionnaire and construct validity were analyzed using Stata (Intercooled Stata version 12 Statacorp, college station, TX, USA). Descriptive data were reported as percentages or as means and SDs for normally and as medians and IQRs for non-normally distributed variables. Statistical significance was set at an alpha level of 0.05 and all tests were two-sided. Differences in means or proportions of variables were assessed using survey commands in Stata to account for clustering. Skewed continuous variables were transformed to improve normality.

#### Construct validity analyses

A comprehensive assessment of each scale’s quality was performed [32]. Item distribution and variation were examined using descriptive analyses. Internal consistency of each construct was examined using Cronbach’s alpha; values of alpha > 0.50 were considered acceptable as *i*) it was a newly developed questionnaire and *ii*) some constructs included only a few items [33]. Repeatability (test-retest) of the questionnaire was examined using the ICC to assess absolute agreement between single items or the sum scores of the constructs; values of ICC > 0.30 were considered to be acceptable.

#### Structural Equation Modeling

Structural Equation Modeling (SEM) was conducted to statistically test the inter-relationships of constructs and their relationship with the four components of dietary behavior in our participant population [34]. SEM is a multivariate technique that allows for the modeling of a series of hypothesized relationships simultaneously. It combines aspects of factor analysis and multiple regression and allows for the inclusion of observed and unobserved (latent) variables (i.e., theoretical constructs) to determine whether the hypothesized associations are consistent with data of the participant population [35, 36]. Prior to modeling the relationship between latent variables, a measurement model was evaluated for each component of dietary behavior. This step involves a confirmatory factor analysis to confirm the relationship between the latent variables (constructs) and their indicator variables (items). The following step, i.e. the testing of the structural model, estimates the strength of the relationships between these latent variables. It also allows for examining the direct and indirect effects among the constructs in the model. Data were examined prior to modeling to ensure they met assumptions of performing a SEM and analyzed using the robust maximum likelihood procedure in LISREL 8.72 [37]. Using multi-level SEM with a small number of clusters (< 100; in our study: 34) and low ICC (<0.25, in our study: < 0.10) has been shown to produce inaccurate results. So even though a cluster sampling design was used, we applied regular SEM [38].

First, correlation coefficients were calculated between the variables of interest. All correlations were <0.70, thus multi-collinearity was not a concern in the present data [39]. Path coefficients were then estimated and the general fit of the model was assessed for each component of dietary behavior. To evaluate the goodness of fit of the model, the χ²-value together with degrees of freedom were calculated, as well as four other indices: the root mean square error of approximation (RMSEA), the normed fit index (NFI), the non-normed fit index (NNFI) and the comparative fit index (CFI). The χ²-value has traditionally been used to test the hypothesis that the relationships suggested in the model provide a plausible explanation of the data, i.e. how well the proposed model structure fits the structure in the observed data set. It is however sensitive to sample size; a large sample size increases the power to reject the models. Other fit indexes have been proposed to compensate for this problem. The RMSEA is a measure of discrepancy between the true population model and the hypothesized model with unknown but optimally chosen parameter estimates. In other words, it favors a more parsimonious model with fewer parameters. It is relatively insensitive to sample size, since it is a population-based index [40]. The CFI and NNFI both compare the fit of the hypothesized model to that of a baseline or null model, where all parameters are assumed to be independent. Values below 0.08 for RMSEA [41] and above 0.90 for NFI, NNFI and CFI [35] indicate an acceptable fit of the data to the hypothesized model. One requirement for using SEM for model testing is complete data with no missing values. To minimize exclusion of observations from the analyses, imputation of missing data values of constructs was performed using the expectation-maximization algorithm (1000 iterations) for those included in SEM analysis [42]. Separate SEM analyses were conducted for each of the dietary behaviors.

## Results

### Participant characteristics

Of the 784 adolescents recruited, *n* = 751 (of which 50.4% were male, and *n* = 594 came from urban areas) were included in the final SEM analysis. Those excluded lacked data on anthropometry (*n* = 5), dietary behavior (*n* = 5), socio-demographic factors (*n* = 5) or on individual/environmental factors (*n* = 18). Excluded adolescents represented 4.2% of the initial sample. They did not differ in terms of mean age (*P* = 0.87) and sex (*P* = 0.81), but excluded adolescents tended to be poorer (*P* = 0.03).

Mean age of the included participants was around 14 years (Table 1); 20.1% of the adolescents were overweight or obese and 4.5% were underweight. More than half of the participants (55%) had low SES; nearly all poor adolescents came from rural areas. BMI was significantly lower in poor adolescents when compared to their peers who were better-off. Age and gender did not differ by SES.

**Table 1:** Participant characteristics.

Variables	Total (*n* = 751)	Poor (*n* = 415)	Better-off (*n* = 336)	*P* value*
Male (%)	50.4	47.2	54.1	0.25
Age (mean y (SD))	13.6 (1.2)	13.7 (1.3)	13.6 (1.1)	0.82
Urban (%)	79.0	64.3	97.3	<0.001
BMI (kg/m²)	20.3 (3.1)	20.0 (2.9)	20.6 (3.3)	0.04

*Differences in means or proportions of variables were assessed using survey commands in Stata to account for clustering.

### Key components of dietary behavior

Median fruit and vegetable intake was limited, while median sugary drink intake was substantial in our population (Table 2). Over one-fifth of daily energy intake came from unhealthy snacking, similar to the E% originating from breakfast. Breakfast intake among the poor was significantly higher and sugary drink intake significantly lower compared to the better-off adolescents. No other differences in dietary behavior by SES were found.

**Table 2:** Key components of dietary behaviour as measured by two 24h dietary recalls.

Dietary behaviour	Total (*n* = 751)		Poor (*n* = 415)		Better-off(*n* = 336)		Difference (Poor—better-off)	*P* value*
	Median	IQR	Median	IQR	Median	IQR		
Fruit (g/d)	121.8	156.6	126.2	154.8	114.6	158.6	11.6	0.83
Vegetables (g/d)	49.7	56.7	50.3	62.2	49.4	50.0	0.9	0.48
Fruit and vegetables (g/d)	180.8	177.0	186.1	175.6	174.4	181.3	11.7	0.48
Sugary drinks (g/d)	150.0	300.0	99.0	237.5	201.6	346.2	–102.6	0.03
Breakfast (E%/d)	21.6	13.6	23.1	13.6	19.4	12.1	3.7	0.002
Unhealthy snacking (E%/d)	22.2	31.0	20.0	30.9	24.6	30.8	–4.6	0.13

*Differences in means or proportions of variables were assessed using survey commands in Stata to account for clustering.

IQR, Inter Quartile Range.

### Construct validity

**Fig 2:**
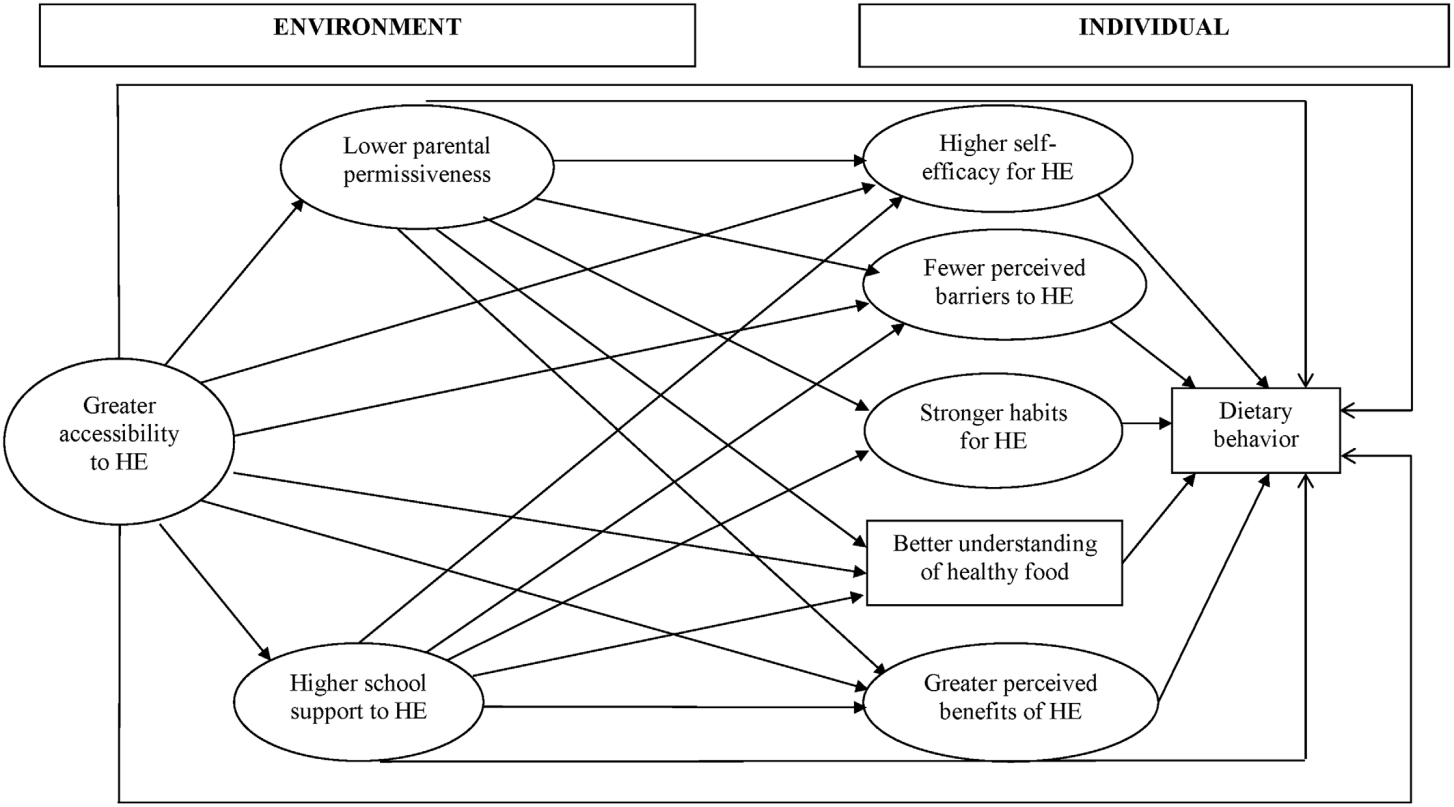
Specification of a SEM for predicting dietary behavior, including mediation effects. Rectangles indicate observed variables, and ellipses latent variables. HE: Healthy Eating.

Internal consistency (Cronbach’s alpha) and repeatability (ICC) were acceptable to good for most of the constructs. Subjective norm, parental rules, parental modeling, school rules and taste did not meet the internal consistency or repeatability criteria, however, and were not used in the model. The retained constructs and their items, ICC and Cronbach’s alpha are presented in Table 3. The resulting framework, which we validated using SEM analysis is shown in Fig 2. For ease of interpretation, constructs are presented as the most favorable conditions for healthy dietary practices.

**Table 3:** Cronbach’s alpha and Intra-class Correlation Coefficient for retained constructs in the model at individual and environmental level.

Constructs	Items	Cronbach’s alpha	ICC sum score
**Individual level**			
Self-efficacy	Suppose you want to eat healthily. How hard is it for you to eat healthy every day?	0.66	0.58
	Suppose you want to eat healthily. How hard is it for you to eat healthy at home?		
	Suppose you want to eat healthily. How hard is it for you to eat healthy at school?		
Attitudinal beliefs			
Perceived benefits	If I eat healthily it helps me to control my body weight	0.64	0.31
	If I eat healthily it makes me feel better		
Perceived barriers	Unhealthy food is cheaper	0.71	0.44
	Healthy food is not available when I am eating		
	Healthy foods don’t taste good		
	I’ve been eating fast food since I was young		
	My parents don’t have time to cook healthy food		
	My body needs unhealthy food		
	Breaks at school are too short to eat healthily		
	I eat unhealthily because I want to eat the same as my friends		
Habit strength	I eat snacks or fast food when I watch TV	0.56	0.46
	I eat snacks or fast food when I go out with friends		
	I eat snacks or fast food when I am going to sports training		
	I eat snacks or fast food when I go on a family trip		
Understanding of what constitutes a healthy food	Eating healthily is eating food without chemicals*	NA	0.22
**Environmental level (home and school environment)**			
Parental permissiveness	My parents let me eat fast food (“Pitty’s”, French fries, hamburgers, etc.) and snacks (ice cream, jelly, candies, etc.) whenever I want to	0.51	0.53
	My parents let me drink sodas whenever I want to		
School support	How often does your school/teachers encourage you to eat healthily?	0.76	0.40
	How often do your teachers/school give you information regarding healthy eating?		
Accessibility to healthy food	My family can’t afford to buy healthy food	0.62	0.47
	There is no weekly market in my neighbourhood		
	Healthy food that is sold around my place is spoiled		
	The places selling healthy food are far from my house		

ICC, Intraclass Correlation Coefficient; NA, Not Applicable.

* when recoded, a better understanding of what constitutes a healthy food was scored as positive for healthy dietary practices.

### Structural equation modeling: conceptual framework

#### Goodness-of-fit of the models

**Fig 3:**
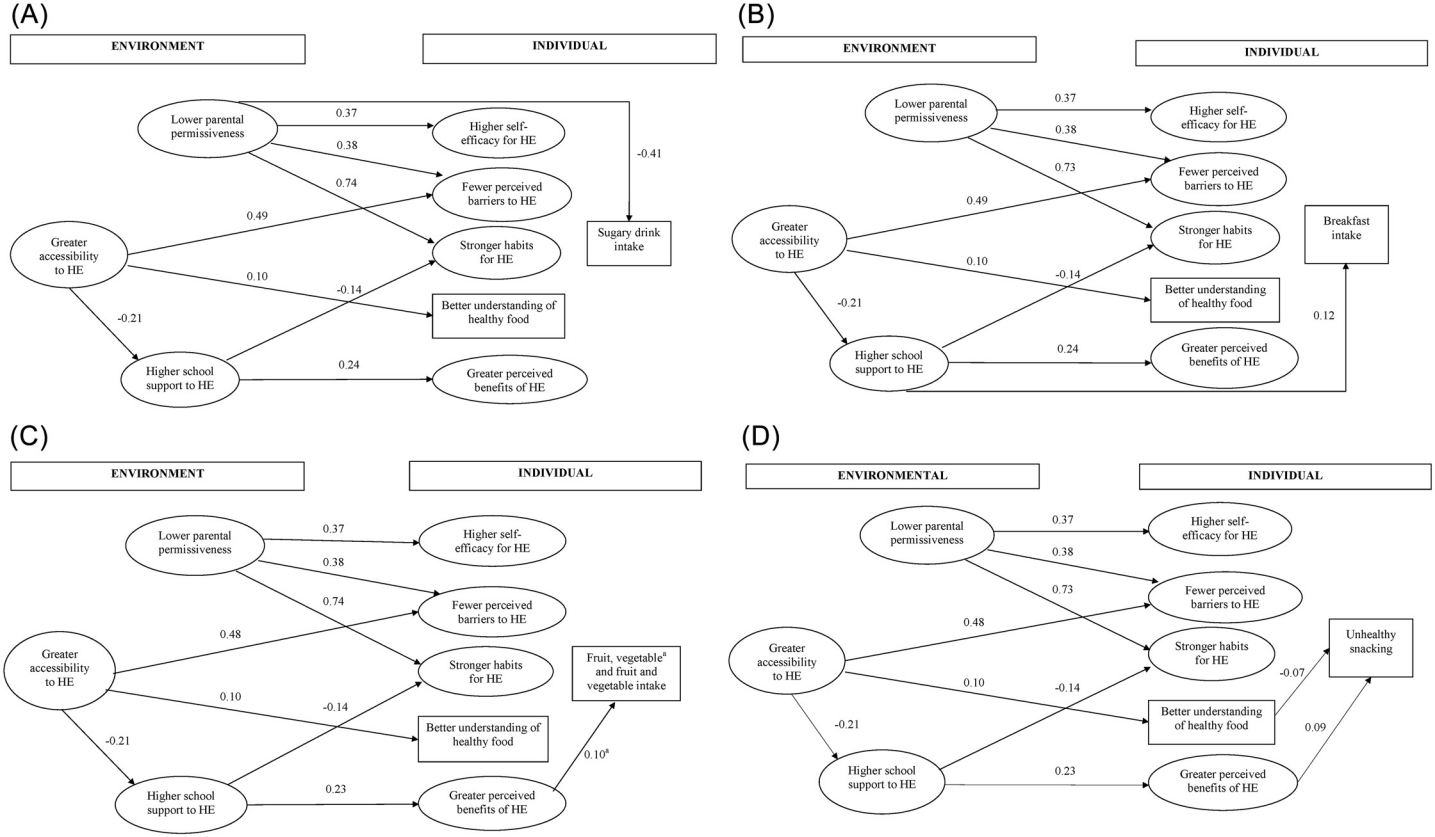
(A) Individual and environmental influences on sugary drink consumption. Only statistically significant paths at P < 0.05 are shown. Goodness of Fit-statistics: χ²(303) = 630.18, p< 0.001; RMSEA = 0.038; NNFI = 0.92; CFI = 0.93, GFI = 0.93. HE: Healthy Eating. (B) Individual and environmental influences on breakfast consumption. Only statistically significant paths at P < 0.05 are shown. Goodness of Fit-statistics: χ²(303) = 630.50, p< 0.001; RMSEA = 0.038; NNFI = 0.92; CFI = 0.93, GFI = 0.94. HE: Healthy Eating. (C) Individual and environmental influences on fruit, vegetable and fruit and vegetable consumption. Only statistically significant paths at P < 0.05 are shown. Goodness of Fit-statistics: χ²(303) = 623.69, p< 0.001; RMSEA = 0.038; NNFI = 0.92; CFI = 0.93, GFI = 0.94. ^a^ significant pathway for vegetable intake only. HE: Healthy Eating(D) Individual and environmental influences on unhealthy snacking. Only statistically significant paths at P < 0.05 are shown. Goodness of Fit-statistics: χ²(303) = 619.19, p< 0.001; RMSEA = 0.037; NNFI = 0.92; CFI = 0.93, GFI = 0.94. HE: Healthy Eating.

The confirmatory factor loadings were significant at the 0.05 level and ranged from 0.40 to 0.86. The overall fit of the measurement model was adequate (χ²(271) = 503,72, p< 0.001; RMSEA = 0.034; NNFI = 0.94; CFI = 0.95, GFI = 0.95). Using these same fit criteria, the overall fit of the structural models predicting fruit, vegetable, fruit and vegetables, unhealthy snacking, sugary drinks, and breakfast intake was also adequate (*See* Fig 3A–3D for details).

#### Interrelationships of the constructs

The majority of the hypothesized inter-relationships of individual and environmental factors were confirmed and appeared to be similar for each of the dietary behaviors **(***See* Fig 3A–3D for details). A strong and expected relationship in the model was the link between greater accessibility to healthy food in the environment and fewer perceived barriers to eating healthily. Greater accessibility was also associated with a better understanding of healthy food, meaning that greater access to healthy foods was related to perceiving healthy foods more in function of their nutritional value rather than their added chemicals (e.g. colorants, chemicals, etc.). Greater accessibility was also inversely associated with higher support from the school to eat healthily, i.e. if healthy food is more accessible, there is less support from the school. Having less permissive (i.e. stricter) parents was directly associated with higher self-efficacy, fewer perceived barriers and stronger habits for healthy eating. The relationship between parental permissiveness and stronger habits for healthy eating was the strongest in the model, suggesting that adolescents with more permissive parents had stronger unhealthy eating habits. Furthermore, greater school support was associated with higher perceived benefits of eating healthily and negatively associated with stronger habits for healthy eating.

#### Sugary drink intake

Lower parental permissiveness was inversely associated with sugary drink intake, indicating that adolescents of more permissive parents consumed more sugary drinks. None of the individual factors were related to sugary drink intake (Fig 3A).

#### Breakfast consumption

School support to healthful eating predicted breakfast consumption, meaning that a more supportive environment at school to eat healthily increased the consumption of breakfast in adolescents. Additionally, the perceived accessibility to healthy foods in the environment was associated with breakfast consumption indirectly through its influence on school support (Fig 3B). This suggests that a lower accessibility to healthy food is related to a more supportive school environment to eat healthily, which is in its turn positively associated with breakfast consumption.

#### Fruit, vegetable, and fruit and vegetable intake

In contrast to both previous models, none of the environmental factors were directly associated with fruit, vegetable or fruit and vegetable intake (Fig 3C). Adolescents perceiving strong benefits of eating healthily ate more vegetables, though significant, the strength of this association was rather low. This association was not significant for fruit intake or fruit and vegetable intake combined. The indirect relationship between greater school support and higher vegetable intake operated through perceived benefits and in its turn, greater school support to eating healthily was influenced by lower accessibility to healthy food. Thus, adolescents’ perception of a greater accessibility to healthy food in the environment indirectly increased their vegetable intake.

#### Unhealthy snacking

Greater perceived benefits of eating healthily were directly associated with more unhealthy snacking, and the indirect relationship between higher accessibility to healthy food and unhealthy snacking was facilitated by greater school support and greater perceived benefits (Fig 3D). The estimated associations indicate that lower accessibility to healthy food is related to more school support to eat healthily and a better understanding of the importance of healthy eating (perceived benefits). Surprisingly, the latter was associated with a higher intake of unhealthy snacks. Furthermore, a better understanding of healthy food was inversely and directly associated with the consumption of unhealthy snacks. This better understanding of healthy food mediated the relationship between the perceived accessibility to healthy foods and consuming unhealthy snacks. Thus, adolescents reporting low access to healthy foods were less likely to believe that the quality of healthy foods is associated with nutritional value, which may explain their higher consumption of unhealthy snacks.

## Discussion

Using SEM, we quantified the relationships between factors and their influence on key components of dietary behavior. The models tested for each of these components performed well, indicating the validity of the conceptual model for our population. These models confirmed the interdependence of factors within and across individual and environmental (physical and social) levels. The fact that these relationships were comparable, both in strength and direction, across behaviors suggests that the interrelationship between environmental and individual factors for dietary behavior were near-identical. How these factors affected the dietary behavior, however, varied by behavior. This finding is in line with previous research documenting that factors affecting dietary behavior differ by type of behavior [19, 43–46].

Only individual level factors (perceived benefits of eating healthily) were found to be directly related to vegetable intake and unhealthy snacking. Interestingly, perceived benefits of eating healthily was directly associated with both increased vegetable and unhealthy snack intake. This somewhat puzzling result may have different explanations. In our adolescent population, choosing to eat healthily (e.g. vegetables) was found to be associated with an untrendy image leading to teasing and marginalization [21]. Eating unhealthy snacks might thus have helped with peer acceptance. In addition, adolescents who are convinced of the benefits of eating healthily and who eat more vegetables, may think they are allowed to also eat unhealthy snacks. Alternatively, it might be that their parents allow them to snack as a reward for eating vegetables [47].

A better understanding of healthy food was a direct and inverse predictor of unhealthy snack intake. Our qualitative work showed that “healthy foods” were often associated with the absence of colorants or other chemical substances rather than with nutritional value of the food [21]. Apparently, this poor understanding of what healthful food entails led to an increased preference for unhealthy snacks. This finding argues for strategies tackling safety risk communication to support healthier food choices.

Only socio-environmental factors were directly associated with sugary drinks and breakfast intake. Having more permissive parents directly and very strongly predicted higher sugary drinks intake, while greater school support for healthful eating directly and strongly predicted higher breakfast intake. Prior studies have similarly reported evidence of parental permissiveness and social support as significant predictors of soft drink and breakfast consumption respectively, in children aged 10–12 years old [48]. In our previous work in this population, adolescents indicated that their parents or school staff had little influence over their food choices [21]. They may thus not want to admit or want to minimize the influence their social environment has on their dietary practices, or they are not consciously aware of it. Furthermore, these results confirm that parents and schools play an important role in some behaviors but not all [21].

We also found that the physical environment, i.e. access to healthy food, was associated indirectly with all dietary behaviors (except for sugary drink intake) and this effect operated through socio-environmental (school support) and individual (perceived benefits of healthy eating, a better understanding of healthy foods) factors. As would be expected, greater accessibility to healthy food increased its consumption (e.g. increased vegetable intake). However, some of the indirect relationships found for accessibility appear more difficult to interpret. The relationships between accessibility and the consumption of breakfast and vegetable intake was mediated through a supportive school environment. Schools supported healthful eating when its access was limited. This finding reflects that they may not see a need to promote and support healthful eating when healthy options are easily available and accessible. The physical environment had no direct impact on any behavior. Thus, one can conclude that individual influences and the social environment are stronger and direct predictors of behavior than the physical environment in our population for certain behaviors. This confirms previous findings from high-income countries that socio-environmental factors are more important than physical environmental factors for healthful eating [19, 49]. As a consequence, simply improving accessibility to healthy food may be insufficient to improve dietary behavior; complementary strategies to address the individual and socio-environmental influences may also be required.

To our knowledge, this is the first study examining the validity of a conceptual model for (multiple components of) dietary behavior in young people from LMICs. This study is unique in the sense that we evaluated a comprehensive conceptual model that was guided by theory and validated using locally collected evidence. Additionally, the individual and environmental factors in the model were constructed meticulously and thus reflect the local context of our adolescent study population. A limitation is that the cross-sectional nature of the data do not allow one to infer causality of the estimated associations. Intervention studies are needed to assess the extent to which changes in factors may lead to actual changes in dietary behavior. Another possible limitation is the use of 24h dietary recall data. This commonly used method may suffer from systematic over- or under-reporting, it depends on the accurate recall of intake and portion size as with all retrospective methods, and may not representing habitual intake. Every attempt was made to collect the highest quality data. Our study enumerators were extensively trained, a food recall kit with locally used household measures was used to assist in portion sizes estimates, and the 24h recall was conducted twice to represent habitual intake. More importantly, random measurement error or a systematic over- or underestimation would not have affected the SEM coefficient estimates reported in this study. Differences in breakfast and sugary drink intake were found between poor and better-off adolescents. We refrained from performing subgroup analyses by SES, however, for two reasons. First, the large majority of better-off adolescents come from urban areas, which means that this variable was as much a proxy for urban/rural residence as it was for SES. Second, we did not have sufficient statistical power to test for differences between subgroups.

## Conclusion

Our finding that dietary behaviors in adolescents are determined by a complex interplay of behaviour-specific individual and environmental factors, has implications for interventions. Interventions targeted to promote healthy dietary practices in our population should develop strategies that i) simultaneously address factors at the individual (e.g. knowledge), social (e.g. school staff and peers) and physical environmental (e.g. accessibility) level; ii) have a multipronged focus in addressing different dietary behaviours. These implications are in line with previous recommendations made for intervention development in LMICs [12].

The extent to which the development of interventions in other LMIC settings need to take culture- and context-related factors into account requires further investigation. This includes research on the validity of evidence- and theory-based models, i.e. investigating the inter-relationships of the influencing factors and their relationship with dietary behavior in LMICs. However, as gathering such evidence is a lengthy and time-intensive process, research to develop valid, feasible and faster ways to map such influencing contextual factors and their relationships with behavior should be a priority. Easy-to-use tools for the development of infant and young child feeding interventions have been developed in the past [50, 51]. These could serve as a guide for the development of tools for the intervention design in adolescent populations in LMICs.

## Abbreviations

LMICs: **Low-and Middle-Income Countries**
SES: **Socio-Economic Status**
BMI: **Body Mass Index**
SD: **Standard Deviation**
IQR: **Inter Quartile Range**
ICC: **Intraclass Correlation Coefficient**
SEM: **Structural Equation Modeling**
RMSEA: **Root Mean Square Error of Approximation**
NFI: **Normed Fit Index**
NNFI: **Non-Normed Fit Index**
CFI: **Comparative Fit Index**
HE: **Healthy Eating**
